# Prevalence of Post–COVID-19 Condition and Activity-Limiting Post–COVID-19 Condition Among Adults

**DOI:** 10.1001/jamanetworkopen.2024.51151

**Published:** 2024-12-13

**Authors:** Anjel Vahratian, Sharon Saydah, Jeanne Bertolli, Elizabeth R. Unger, Cria O. Gregory

**Affiliations:** 1 Division of Health Interview Statistics, National Center for Health Statistics, Centers for Disease Control and Prevention, Hyattsville, Maryland; 2 National Center for Immunization and Respiratory Diseases, Centers for Disease Control and Prevention, Atlanta, Georgia; 3 National Center for Emerging and Zoonotic Infectious Diseases, Centers for Disease Control and Prevention, Atlanta, Georgia

## Abstract

This cross-sectional study examines the prevalence of ever and current post–COVID-19 condition (long COVID) and self-reported limitations of activity due to symptoms of post–COVID-19 condition among US adults.

## Introduction

Data from the 2022 National Health Interview Survey (NHIS)^1^ showed that 6.9% of adults in the US ever had post–COVID-19 condition (PCC; also known as long COVID), defined as the presence of symptoms lasting 3 months or longer that they did not have before having COVID-19. Nearly one-half of them (3.4% of all adults) had PCC at the time of interview. In 2023, NHIS included a new question for those with PCC to ascertain the degree to which these long-term symptoms reduce their ability to carry out day-to-day activities compared with a time prior to having COVID-19. This cross-sectional analysis uses these new data to describe the prevalence of PCC (ever and current) and self-reported limitations of activity due to symptoms of PCC.

## Methods

Data from the 2023 NHIS adult interview were used for this cross-sectional analysis.^2^ NHIS is a nationally representative household survey of the civilian noninstitutionalized population residing in the US. This well-established, long-running cross-sectional study is conducted annually by the National Center for Health Statistics (NCHS). The 2023 NHIS was approved by the NCHS Ethics Review Board. Respondents provided verbal consent. The results presented adhere to STROBE reporting guidelines.

One adult was randomly selected from each eligible household to answer more detailed questions about their health. In 2023, the adult was asked whether they ever had COVID-19. If yes, they were asked whether they had any symptoms lasting 3 months or longer that they did not have before having COVID-19 and, if so, whether they had symptoms now. If yes, they were asked how much these long-term symptoms reduce their ability to carry out day-to-day activities compared with the time before they had COVID-19. For this analysis, any activity limitation (responses of “a little” or “a lot” rather than “not at all”) was defined as having activity-limiting PCC, and all estimates were calculated to be reflective of all adults to allow for a population prevalence.

Analyses were conducted using SAS-callable SUDAAN software version 11.0 (SAS Institute) to account for the complex sample design of the NHIS.^2^ All estimates were based on self-report, weighted to be nationally representative, and met NCHS Data Presentation Standards for Proportions.^3^ Hispanic origin and race were asked as separate questions, and race was based on the adult’s description of his or her own racial and ethnic identity.^2^ Data on race and ethnicity were included to estimate whether differences were observed across groups. Urbanization level was drawn from the 2013 NCHS Urban-Rural Classification Scheme for Counties.^4^ Family income was imputed when missing.^5^ *P* values were calculated using χ^2^ tests for nominal categorial variables and trend tests using orthogonal polynomials for ordinal categorical variables. Statistical significance for all estimates was defined as a 2-sided *P* < .05.

## Results

In 2023, among 29 522 respondents (NHIS response rate, 47.0%), 8.4% (95% CI, 8.0%-8.8%) of adults in the US reported they ever had PCC, 3.6% (95% CI, 3.3%-3.9%) currently had PCC, and 2.3% (95% CI, 2.1%-2.5%) currently had activity-limiting PCC (Table). Significant differences across all 3 outcomes were observed by sex, sexual orientation, age, race and Hispanic origin, family income, and urbanization. The percentage of adults who ever had PCC, currently had PCC, and currently had activity-limiting PCC decreased with increasing family income. The prevalence of these 3 outcomes also increased with increasing rurality of the place of residence. Among adults who currently had PCC, 64.5% experienced symptoms that were activity limiting (data not shown).

**Table.** Characteristics of Adults Who Ever Had PCC, Currently Had PCC, and Had Activity-Limiting PCC

Characteristic	Ever had PCC (n = 2398)		Currently had PCC (n = 1063)		Had activity-limiting PCC (n = 706)	
	Percentage (95% CI)	*P* value	Percentage (95% CI)	*P* value	Percentage (95% CI)	*P* value
Overall	8.4 (8.0-8.8)	NA	3.6 (3.3-3.9)	NA	2.3 (2.1-2.5)	NA
Sex						
Women	10.2 (9.6-10.8)	<.001	4.6 (4.2-5.0)	<.001	3.1 (2.8-3.4)	<.001
Men	6.4 (5.9-6.9)		2.5 (2.2-2.9)		1.5 (1.2-1.8)	
Sexual orientation						
Gay or lesbian	9.3 (6.4-13.0)	.002	4.8 (2.8-7.6)	.007	3.6 (1.8-6.3)	.006
Straight	8.2 (7.8-8.7)		3.5 (3.2-3.8)		2.2 (2.0-2.4)	
Bisexual	14.0 (11.1-17.4)		6.8 (4.8-9.2)		5.7 (3.8-8.0)	
Age, y						
18-34	8.1 (7.4-9.0)	<.001^a^	2.8 (2.4-3.4)	<.001^a^	1.7 (1.3-2.1)	<.001^a^
35-49	9.9 (9.0-10.8)		4.1 (3.5-4.8)		2.5 (2.0-3.0)	
50-64	9.4 (8.6-10.2)		4.4 (3.9-5.0)		2.9 (2.5-3.4)	
≥65	5.9 (5.4-6.5)		3.1 (2.8-3.6)		2.3 (2.0-2.7)	
Race and Hispanic origin						
Hispanic	9.4 (8.4-10.5)	<.001	3.8 (3.1-4.6)	<.001	2.4 (1.8-3.0)	.002
Non-Hispanic American Indian and Alaska Native	12.6 (8.2-18.1)		6.3 (3.3-10.7)		NA^b^	
Non-Hispanic Asian	4.4 (3.4-5.7)		1.8 (1.1-2.8)		1.2 (0.6-2.0)	
Non-Hispanic Black	6.6 (5.6-7.7)		2.7 (2.0-3.4)		1.8 (1.4-2.4)	
Non-Hispanic White	8.7 (8.2-9.2)		3.9 (3.5-4.2)		2.5 (2.2-2.7)	
Non-Hispanic other and multiple races^c^	9.6 (6.8-13.1)		3.7 (2.2-5.8)		3.1 (1.7-5.1)	
Family income, % of Federal Poverty Level						
<100	9.6 (8.3-11.1)	.006^d^	4.3 (3.4-5.3)	.003^d^	3.2 (2.5-4.1)	<.001^d^
100 to <200	9.0 (8.0-10.1)		4.5 (3.8-5.3)		3.4 (2.8-4.1)	
200 to <400	8.6 (7.9-9.4)		3.6 (3.1-4.1)		2.1 (1.7-2.5)	
≥400	7.6 (7.0-8.2)		3.1 (2.7-3.5)		1.8 (1.5-2.1)	
Urbanization level						
Large central metropolitan	8.0 (7.3-8.7)	.03^a^	3.1 (2.6-3.6)	<.001^d^	2.0 (1.7-2.4)	.001^d^
Large fringe metropolitan	7.3 (6.6-8.1)		2.9 (2.5-3.5)		1.9 (1.5-2.3)	
Small and medium metropolitan	8.8 (8.0-9.6)		4.1 (3.6-4.7)		2.7 (2.3-3.2)	
Nonmetropolitan	10.0 (9.0-11.2)		4.8 (4.0-5.6)		2.9 (2.3-3.5)	

Abbreviations: NA, not applicable; PCC, post–COVID-19 condition.

^a^ Significant by quadratic test of trend.

^b^ Estimate did not meet National Center for Health Statistics Data Presentation Standards for Proportions.^3^

^c^ Includes those who did not identify as American Indian and Alaska Native, Asian, Black, or White single race, Hispanic, and those who identified as more than 1 race.

^d^ Significant by linear test of trend.

## Discussion

In this cross-sectional study, updated national prevalence estimates of PCC (ever and current) and new estimates of activity-limiting PCC are provided. A limitation of this work is that the data were from self-reports and were not confirmed by medical evaluation. This work supports the Department of Health and Human Services efforts to assess the overall disease burden of PCC across the US population.^6^

## Notes

Supplement.
Data Sharing Statement
