# Supplementary material for: Prevalence of Post–COVID-19 Condition and Activity-Limiting Post–COVID-19 Condition Among Adults
Source: JAMA Netw Open. 2024 Dec 13;7(12):e2451151. doi: 10.1001/jamanetworkopen.2024.51151 (PMC11645641; doi:10.1001/jamanetworkopen.2024.51151)
Supplement: Supplement. — Data Sharing Statement [file jamanetwopen-e2451151-s001.pdf]

Data Sharing Statement

Vahratian. Prevalence of Post–COVID-19 Condition and Activity-Limiting Post–COVID-19 Condition Among Adults. *JAMA Netw Open*. Published December 13, 2024.
doi:10.1001/jamanetworkopen.2024.51151

Data
Data available: Yes
Data types: Deidentified participant data
How to access data: https://www.cdc.gov/nchs/nhis/2023nhis.htm
When available: beginning date: 07-18-2024

Supporting Documents
Document types: None

Additional Information
Who can access the data: Data are publicly available.
Types of analyses: Data are publicly available
Mechanisms of data availability: Data are publicly available
